# Prevalence, patterns, and attitude regarding dietary supplement use in Saudi Arabia: Data from 2019

**DOI:** 10.1371/journal.pone.0274412

**Published:** 2022-09-21

**Authors:** Anwar M. Alhashem, Rawan A. Alghamdi, Rawan S. Alamri, Wejdan S. Alzhrani, Maha S. Alrakaf, Njoud A. Alzaid, Abeer S. Alzaben

**Affiliations:** Department of Health Sciences, College of Health and Rehabilitation Sciences, Princess Nourah Bint Abdulrahman University, Riyadh, Saudi Arabia; PLOS (Public Library of Science), UNITED KINGDOM

## Abstract

Dietary supplements are products containing dietary elements including vitamins, minerals, amino acids, herbs, or botanicals. They can aid consumers with low dietary intake and quality, as well as those with high demands, by boosting nutritious value. A cross-sectional study was conducted among adults living in Saudi Arabia aged between 18–60 years old using online self-administered questionnaire. Information regarding sociodemographic characteristics, use and type of dietary supplements, and attitude toward and patterns of dietary supplement use was collected. The chi-square test, Pearson correlations, and the independent t-test were used. In total, 531 participants (115 men and 416 women) were included. Approximately half of the participants (51.8%, n = 275) used dietary supplements. Participants who were using dietary supplements were significantly younger (29.16 ± 9.32 years), more highly educated (85.5%, n = 235), and worked in the health sector (63.7%, n = 100). Herbal supplement use was associated with older age and female sex. Single mineral supplements were used more frequently by married, female, pregnant, or breastfeeding participants and those not working in the health sector. Fatty supplements were used more frequently by participants with a higher level of education. Regarding the attitude toward dietary supplement use, women, single participants, and health care workers showed a significant positive attitude. In-depth investigation into the amount of and reasons for dietary supplement use in the health sector is required. Additionally, educating pregnant and breastfeeding women on the importance of dietary supplements is necessary.

## Introduction

Dietary supplements are defined as products containing dietary ingredients such as vitamins, minerals, amino acids, herbs, or botanicals [1]. By adding nutritional value, they can be beneficial to individuals with poor dietary intake and quality as well as those with high requirements [2]. The prevalence of dietary supplement use varies among regions, ranging from 50% in the United States to 71% in Denmark [3–10]. Moreover, different variables have been associated with their use, including age, female sex, and education level [3, 8–10]. Studies have shown that excess use of dietary supplements may result in serious health risks, such as toxicity, contamination with heavy metal ions, bleeding, nausea, diarrhea, and stomach discomfort, and liver failure [1, 2, 11, 12]. A comprehensive examination should ideally include a full medical and nutritional history, food evaluation, as well as biochemical tests [2].

Limited studies have been conducted in the Arab region to measure the prevalence of dietary supplement use. The prevalence among students in the United Arab Emirates (UAE) was 39% in 2015 [5], while that among students in Jordan was 27.4% in 2005 [4]. Education level and family income were shown to be correlated with dietary supplement intake in the Arab region [4–7]. Moreover, the majority of studies showed that female sex was associated with dietary supplement use, possibly because of the high risk of deficiency, decreased dietary intake, or an increase in requirements during pregnancy [3–5, 8–10].

In Saudi Arabia, data on the frequency, trend, and attitude toward dietary supplement usage are still insufficient [6, 7]. This study aimed to assess the prevalence, patterns, and attitude regarding dietary supplement use in Saudi Arabia, as well as their relationships with sociodemographic factors.

## Materials and methods

### Participants

Saudi Arabia’s current population is 35,715,310 as of the first quarter of 2022, with the proportion of female residents being 42.17%. Saudi Arabia has a median age of 31.8 years. Saudi Arabia has a population density of 16 persons per square kilometer, and 84.0% of the population lives in cities. Riyadh, the capital, has by far the most residents, with 4,205,961 individuals [13]. A community-based descriptive cross-sectional study measuring the prevalence of dietary supplement use was conducted between January and April 2019 in Saudi Arabia, using an online self-administered questionnaire. The questionnaire was distributed in Arabic language via the WhatsApp messaging app using the snowball sampling technique. The number of sample size is 385 participants with a confidence level of 95% and a confidence interval of 5%. These numbers were obtained by using a sample size calculator (X = Zα/22 *p*(1-p)/MOE2). In this equation, Zα/2 represents the critical value of the normal distribution for a confidence level of 95%, with an α of 0.05 and a critical value of 1.96. MOE represents the margin of error and p represents the sample proportion. The sampling stopped when it reached 500 participants to clean the research data easily. The research was conducted at the Clinical Nutrition Department, Princess Nourah University, and the study protocol was approved by our Institutional Review Board (18–0379). Written informed consent was obtained from all participants through the questionnaire. Saudi adults aged 18–60 years who are living in Saudi Arabia were included. Dietary supplement use was defined as any intake in the previous 12 months, as described in a previous study [3]. Supplement users were defined as individuals who used at least one of the specified dietary supplements in the previous 12 months [7]. The questionnaire was translated into Arabic and back translated into English. It was divided into two sections.

### Sociodemographic characteristics, use, and type of dietary supplements

Sociodemographic information was collected, including age, sex, marital status, pregnancy and breastfeeding status, presence of chronic illnesses, occupation (health vs. non-health sectors), education level, monthly family income, and region. Details regarding the use and type of dietary supplement were based on a previous study [7].

### Attitude toward and patterns of dietary supplement use

The former was assessed using three closed-ended questions regarding the attitude toward using dietary supplements with or without medical advice, side effects, and undergoing laboratory testing prior to supplementation. The attitude toward using dietary supplements questions were adapted from Alfawaz et al. (2017) [7]. The latter was assessed using four closed-ended questions regarding the use of supplements based on medical prescriptions, the frequency of supplement use, reading the attached instructions, and the reason for dietary supplementation.

### Data analysis

Statistical analyses were performed using IBM SPSS Statistics for Macintosh (version 26.0; Armonk, NY). Categorical data are presented as frequencies and percentages for the whole sample, supplement users, and nonusers, whereas continuous data are presented as mean (± SD) or median. The chi-square test was used to determine whether the users differed from nonusers in terms of categorical sociodemographic variables. Pearson correlations were used to assess the relationship between sociodemographic factors and dietary supplement intake. Independent t-tests were used to determine whether the continuous sociodemographic variables were associated with dietary supplementation for measures using an interval scale. Logistic regression analysis for measures using an ordinal scale such as herbal and protein intake across sex and protein intake across marital status. Differences were considered statistically significant at *P* <0.05. The supplements were grouped and labeled into seven groups which was adapted from Baltazar-Martins et al. (2019) [14].

1. Herbal supplements
2. Multivitamins and/or multiminerals
3. Single preparation of vitamins, such as beta carotene or vitamins A, B12, B9, B complex, C, D, and E
4. Single preparation of minerals, such as calcium, zinc, iron, magnesium, and potassium
5. Fatty acids, including omega 3, and cod liver oil
6. Proteins in the form of powders, bars, and shakes
7. Others, which include coenzyme Q10 and any other supplements not related to the above categories

## Results

### The demographic characteristics of the study participants

Table 1 presents the sociodemographic variables of the entire cohort, supplement users, and nonusers. In total, 531 participants were included in the study, with a mean age of 30.17 ± 10.25 years and a median age of 27 years. Approximately half of the participants (51.8%, n = 275) consumed dietary supplements.

**Table 1:** Frequencies and percentages of sociodemographic variables by dietary supplement use (n = 531).

Variables n (%)	All participants (n = 531)	Supplement users (n = 275)	Supplement nonusers (n = 256)	*P-*value
Sex				
Female	416 (78.3%)	223 (53.6%)	193 (46.4%)	0.11
Male	115 (21.7%)	52 (42.5%)	63 (54.8%)	
Marital status				
Single	335 (44.3%)	132 (56.2%)	103 (43.8%)	0.16
Married	276 (52.0%)	136 (49.3%)	140 (50.7%)	
Divorced/Widowed	20 (3.8%)	7 (35.0%)	13 (65.0%)	
Pregnant or breast feeding				
Pregnant	23 (5.5%)	18 (78.3%)	5 (21.7%)	0.03*1
Breastfeeding	24 (5.7%)	10 (41.7%)	14 (58.3%)	
Neither	374 (88.8%)	197 (52.7%)	177 (47.3%)	
Chronic illness				
No	479 (90.2%)	246 (51.4%)	233 (48.6%)	0.55
Yes	52 (9.8%)	29 (55.8%)	23 (44.2%)	
Education				
Less than university	109 (20.5%)	40 (14.6%)	69 (63.3%)	0.001**2
University and above	422 (79.5%)	235 (85.5%)	187 (44.3%)	
Occupation sector				
Health	157 (36.6%)	100 (63.7%)	57 (36.3%)	0.009**
Non-health	272 (63.4%)	138 (50.7%)	134 (49.3%)	
Income				
Less than 5,000 SR3	76 (14.3%)	37 (48.7%)	39 (51.3%)	0.88
5,000 to 10,000 SR	143 (26.9%)	72 (50.3%)	71 (49.7%)	
10,000 to 16,000 SR	128 (24.1%)	68 (53.1%)	60 (46.9%)	
More than 16,000 SR	184 (34.7%)	98 (53.3%)	86 (46.7%)	
Region				
North	103 (19.4%)	57 (55.3%)	46 (44.7%)	0.55
South	75 (13.6%)	40 (55.6%)	32 (44.4%)	
East	67 (12.6%)	34 (52.2%)	32 (47.8%)	
West	103 (19.4%)	46 (44.7%)	57 (55.3%)	
Middle	186 (35.0%)	97 (52.2%)	89 (47.8%)	

^1^Saudi Riyals

* *P* < 0.05

** *P* < 0.01

** *P* < 0.001

The mean age differed significantly between the two groups (*t* [500] = 2.34; *P* = 0.02). Participants who used dietary supplements were significantly more likely to be younger than those who did not (29.16 ± 9.32 years vs. 31.25 ± 11.08 years). A difference was also found in the occupation, as those who worked in the health sector were significantly more likely to use dietary supplements than were those working in other fields (χ^2^ (1, *N* = 421) = 6.77; *P* = 0.009).

In terms of pregnancy and breastfeeding status, the dietary supplement users and nonusers differed significantly (χ^2^ [2, *N* = 421] = 7.12; *P* = 0.03). The percentage of pregnant women using dietary supplements was greater than the percentages of those who were breastfeeding (78.3%, n = 18 vs. 41.7%, n = 10; *P* = 0.02) and those who were neither pregnant nor breastfeeding (52.7%, n = 197; *P* = 0.01). The percentage of breastfeeding women using dietary supplements did not significantly differ from that of those who were neither pregnant nor breastfeeding. Moreover, a difference in education level was noted (χ^2^ [1, *N* = 421] = 12.51; *P* <0.001). Participants who completed university education were more likely to use dietary supplements than were those who did not complete university education (85.5%, n = 235 vs. 14.6%, n = 40).

### Patterns of dietary supplement use

**Fig 1:**
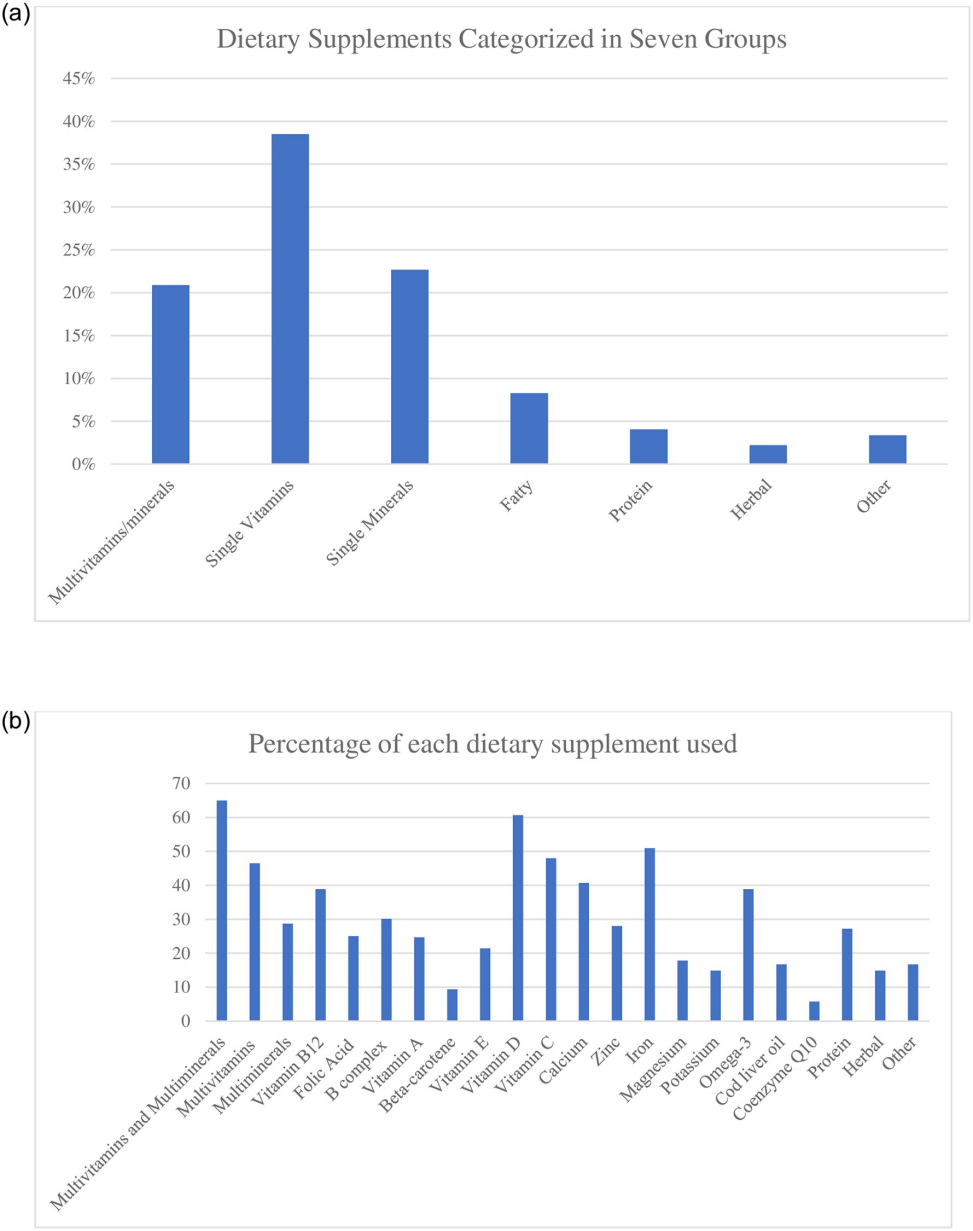
a. The percentage of participants using dietary supplement, categorized in seven groups (n = 275). b. The percentage of participants using each dietary supplement (n = 275).

Due to the wide variety of dietary supplements, this study categorized them into seven groups. (1) Herbal supplements, (2) Multivitamins and/or multiminerals, (3) Single preparation of vitamins, such as beta carotene or vitamins A, B12, B9, B complex, C, D, and E, (4) Single preparation of minerals, such as calcium, zinc, iron, magnesium, and potassium, (5) Fatty acids, including omega 3, and cod liver oil, (6) Proteins in the form of powders, bars, and shakes, (7) Others, which include coenzyme Q10 and any other supplements not related to the above categories. Fig 1a shows the percentage of dietary supplements categorized into seven groups. Fig 1b displays the percentage of each dietary supplement.

In terms of dietary supplement intake, the highest possible score for each was reflected in the highest value in the range column. According to Fig 1a, the supplement that most participants used was a single vitamin. As indicated in Fig 1b, the supplement that was taken by the majority of participants was a multivitamin and/or multimineral by 65% of the users. Additionally, 60.7% of the participants used vitamin D, and more than half of the participants used iron.

**Fig 2:**
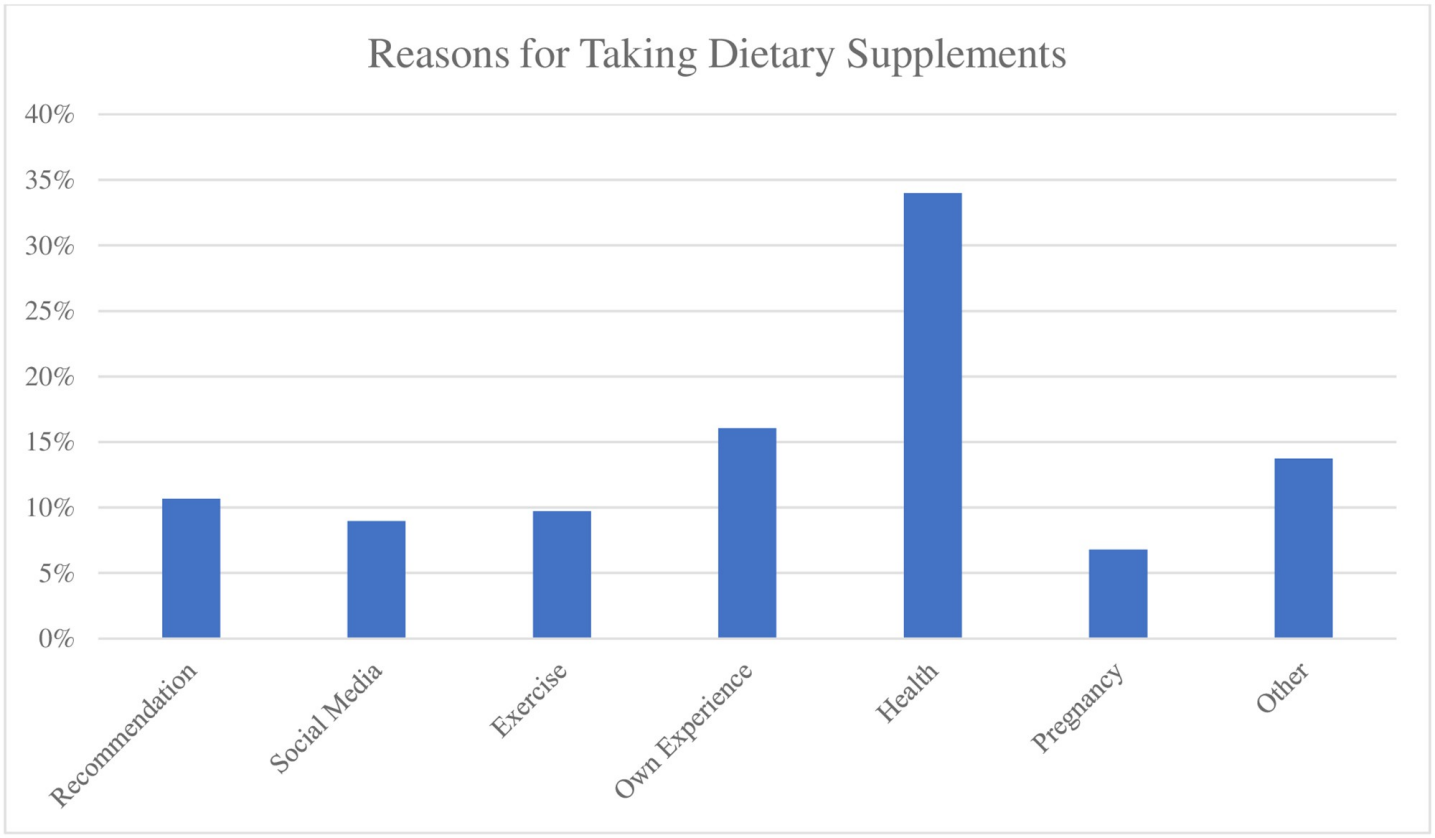
The participants’ reasons for using dietary supplements (n = 275).

According to the reasons for using dietary supplements, 80% of the participants cited health maintenance, whereas 38% mentioned personal experience. In addition, 23% of the participants began using them based on recommendations from friends or family, and 25% of female participants due to pregnancy or lactation. The reasons for using dietary supplements differed between pregnant or breastfeeding women and those in neither category (*t* (223) = 3.12; *P* = 0.002), with the former group providing significantly more reasons than the latter (6.75 ± 1.55 vs. 4.35 ± 1.18). One-third of the participants were using dietary supplements on the basis of information they gathered from the internet, television, or social media, 23% were using them to maintain their gym and fitness regimes, and 32% were using them for other reasons. Participants mentioned an average of six reasons for using dietary supplements, as shown in Fig 2. In addition, 65 participants used some or all of their dietary supplements based on the prescription of a physician, and more than half read the attached instructions prior to use.

### Association between sociodemographics and different types of dietary supplements

Table 2 present the significant associations between sociodemographics variables and types of dietary supplements. *Age*: Age was only significantly correlated with the use of herbal supplements (*r* = 0.15; *P* = 0.01). The older the participant, the greater their possibility of using herbal supplements.

**Table 2:** Significant association between sociodemographics and different types of dietary supplements.

Sociodemographics	Types of dietary supplements	*Tests*	*P*-value
Older age	Herbal	*r* = 0.15	0.011*
Women	Single minerals	*t*(273) = 2.76	0.006**
Marital status	Single minerals	*t*(273) = -2.42	0.016*
Single status	Protein	Wald = 7.32	0.01**
Pregnant or breastfeeding	Single minerals	*t*(41) = 3.17	0.0003***
Undergraduate and graduate degrees	Fatty supplements	*t*(56) = -2.11	0.040*
Working in non-health occupation sector	Single minerals	*t*(236) = -2.39	0.017*
Earning between 10,000–16,000 SR	Single minerals	*F*(3, 271) = 2.91	0.035*

* *p* < .05.

** *p* < .01.

*** *p* < .001.

*r* = correlation, *t = t-test*, *F = Levene’s test*, *Wald = wald test*

#### Sex

Sex was significantly related to the intake of single minerals (*t* (273) = 2.76; *P* = 0.006). Women used significantly more single minerals than did their male counterparts (1.46 ± 1.38 vs. 0.87 ± 1.47). There was also a difference regarding herbal and protein supplement intake. Women were 2.67 times more likely to use herbal supplements (95% CI: 1.29–5.57), and men were 4.85 times more likely to use protein supplements (95% CI: 2.57–9.17).

#### Marital status

Marital status was significantly related to the intake of single minerals (*t* (273) = -2.42; *P* = 0.016). Married participants were more likely to use single minerals than were their unmarried counterparts (1.55 ± 1.37 vs. 1.14 ± 1.43). Moreover, marital status was significantly associated with protein intake. Table 3 presents the logistic regression results for herbal and protein intake across sex and for protein intake across marital status. Married participants were 0.47 times more likely to use herbal supplements than were unmarried ones (95% CI: 0.27–0.81).

**Table 3:** Logistic regression results for herbal and protein intake across sexes (N = 275).

Variables	*B*	*SE*	Wald	*df*	*OR*	95% CI for *OR*	
						Lower	Upper
Herbal supplement							
Sex	0.98	0.37	6.92**	1	2.67	1.29	5.57
Protein supplement							
Sex	1.58	0.33	23.6***	1	4.85	2.57	9.17
Marital status	-0.76	0.28	7.32**	1	0.47	0.27	0.81

** *p* < .01.

*** *p* < .001.

#### Pregnancy and breastfeeding status

Pregnancy and breastfeeding status was significantly related to the intake of single minerals (*t* (41) = 3.17; *P* < 0.001). Pregnant or breastfeeding participants used more single minerals than did their counterparts (2.11 ± 1.10 vs. 1.38 ± 1.41).

#### Chronic health status

There were no significant differences between those with chronic and non-chronic diseases.

#### Education

Since there were only a few participants with less than a university degree, they were all analyzed in the same group. Education level was significantly related to the intake of fatty supplements (*t* (56) = -2.11; *P* = 0.04). Participants with a university degree or higher used significantly more fatty supplements than did those without such education (0.59 ± 0.72 vs. 0.35 ± 0.66).

#### Occupation sector

Occupation was significantly related to the intake of single minerals (*t* (236) = -2.39; *P* = 0.02). Those who did not work in the health sector used significantly more single minerals than did their counterparts (1.50 ± 1.42 vs. 1.06 ± 1.37).

#### Income

Income was significantly related to the intake of single minerals (*F* (3, 271) = 2.91; *P* = 0.04). Post-hoc Tukey comparisons indicated that those earning between 10,000 and 16,000 Saudi Riyals per month used significantly more single minerals than did those earning more than 16,000 Saudi Riyals (1.62 ± 1.49 vs. 1.02 ± 1.27; *P* = 0.04).

#### Region

There were no significant differences across regions.

Table 3 presents the logistic regression results for herbal and protein intake across sex and for protein intake across marital status. Married participants were 0.47 times more likely to use herbal supplements than were unmarried ones (95% CI: 0.27–0.81).

### Attitude toward dietary supplements

**Fig 3:**
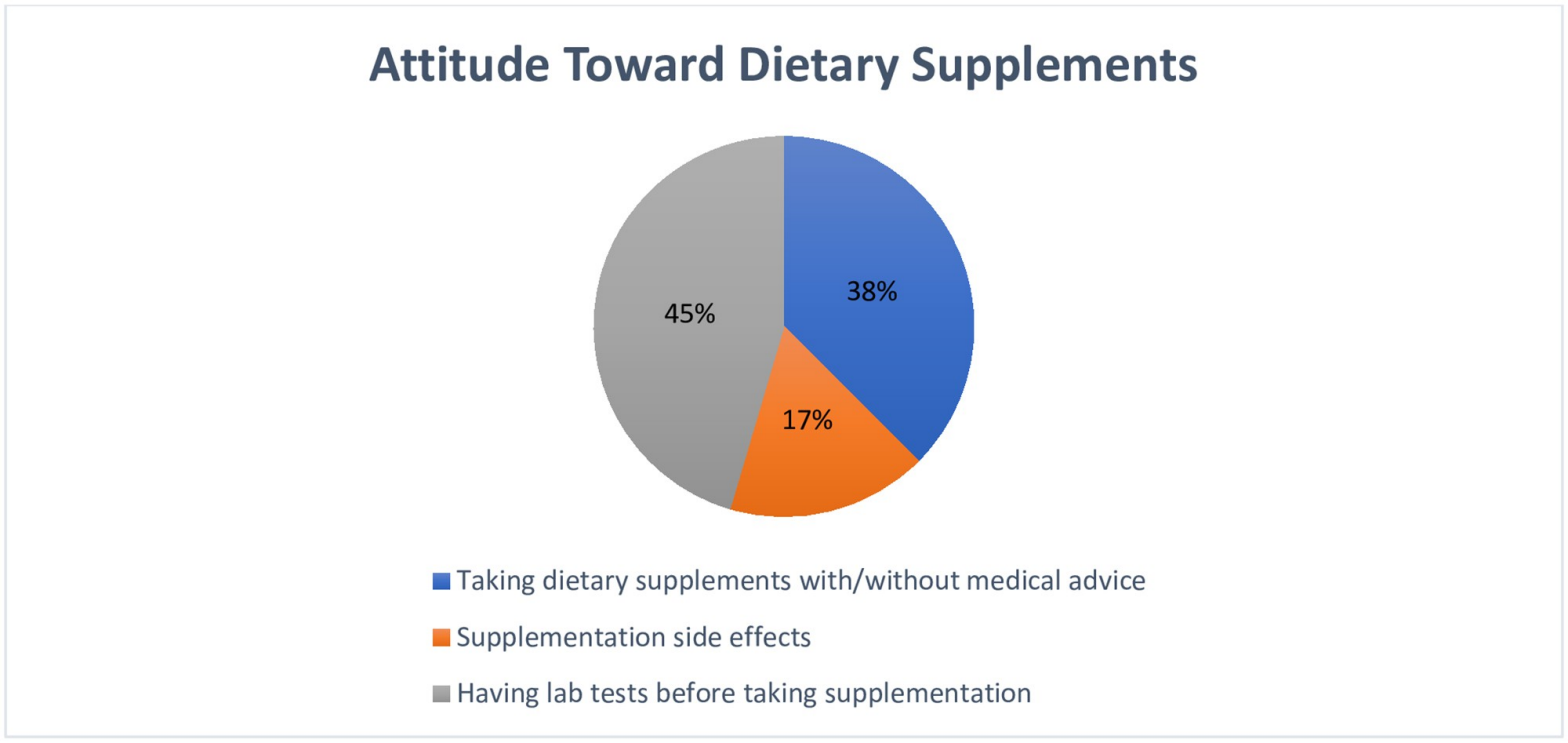
The attitude of participants toward dietary supplement use (n = 275).

Three items were used to measure the attitude toward dietary supplements. Higher scores, the highest possible score being 6, indicated a more positive and better attitude toward dietary supplement use (4.51 ± 1.09). The majority of the participants were aware of the difference between using dietary supplements with and without medical consultation and knew the importance of undergoing a laboratory test prior to using them. About 65% were aware of the negative side effects of dietary supplementation. Participants’ agreement with these items is shown in Fig 3.

### Association between sociodemographics and attitude toward dietary supplements

Table 4 presents the significant associations between sociodemographic variables and attitude toward dietary supplements.

**Table 4:** Significant association between sociodemographics and attitude toward dietary supplements.

Sociodemographics	Positive Attitude	Association Tests	*P-*value
Sex	Female	*t* (273) = 2.05	0.04*
Marital status	Single status	*t* (262) = 2.47	0.01**
Occupation sector	health workers	*t* (236) = 2.59	0.01**

#### Age

Age was not significantly associated with attitude.

#### Sex

Men and women differed in terms of their attitude toward using dietary supplements (*t* (273) = 2.05; *P* = 0.04). Women were more likely to have a positive attitude toward dietary supplements than were men (4.57 ± 1.04 vs. 4.23 ± 1.28).

#### Marital status

Since there were only a few divorced and widowed participants, they were all analyzed in the same group. Single and married participants differed in terms of their attitude toward using dietary supplements (*t* (262) = 2.47; *P* = 0.01). Single participants expressed a more positive attitude toward using dietary supplements than did married ones (4.67 ± 0.98 vs. 4.35 ± 1.18).

#### Occupation sector

In terms of attitude toward dietary supplementation, participants who worked in the health sector differed from those working in other sectors (*t* (236) = 2.59; *P* = 0.01). The findings from the independent t-tests showed that health sector workers had a significantly more positive attitude than did their counterparts (4.74 ± 0.84 vs. 4.41 ± 1.16).

## Discussion

This study aimed to assess the prevalence, patterns, and attitude regarding dietary supplement use in Saudi Arabia in 2019.

Almost 47% of Saudis used at least one supplement in 2019. National data from the United States and Australia showed that the prevalence of dietary supplement use in adults was approximately 50% in 2011 and 23.5% in 2014–2015, respectively [15, 16]. Other studies showed higher prevalences in countries such as Denmark, where the prevalence was 71%, and in the United States [3, 6–10, 17]. Compared with the prevalence in Arab countries, that among students in Jordan and UAE was 27% and 39%, respectively [4, 5].

In our study, almost half of the participants were using dietary supplements. This information is alarming because the Saudi population may be unaware of the adverse effects of dietary supplement overuse. This finding was reported in different populations [17–19].

Data regarding the prevalence, patterns, and attitude regarding dietary supplement use remain insufficient in Saudi Arabia. Six previous cross-sectional studies have been conducted [6, 7, 20–23] to this purpose. The first found that, in eastern Saudi Arabia, 45% of female medical students used dietary supplements [6], while the second study reported 77% in a survey of female students living in Riyadh [7]. Other studies showed that the disparities vary from 30% to 76% [7, 19–23]. The disparity between these studies might be related to differences in the study design, demographics of participants (pregnant women, university students, female universities, and athletes, etc.), age, sex, place of residence, and education level [7, 20–23]. In order to plan national awareness programs, studies with large sample sizes are required for assessment. In addition, there is no clear global definition for supplement use. Nonetheless, our results were similar to the national results from the United States [16].

A younger age, university degree education and higher, health sector workers, and pregnancy were shown to be factors influencing the use of dietary supplements. This is similar to the results of a study by Algaeed et al., where they found that approximately 74% of consumers were less than 33 years old, and more than 74.4% had a bachelor’s degree or higher education [22]. A similar article by Alowais et al. found that 84.5% of dietary supplement users in Saudi Arabia were college graduates, and almost 60% of the participants were less than 25 years old. The association between education level, occupation sector, and dietary supplement use has been reported in various studies [5–7]. Our study supports the above findings showing a significant association between level of education and dietary supplement use in the medical sector. Although overuse has been reported among older individuals and long-term home residents, it has not been well examined, especially in the higher education and medical sectors [24].

In general, women are more likely to use dietary supplements and herbs than are men [15, 25]. This is mainly due to pregnancy or wanting to maintain healthy hair and skin [7, 21]. In addition, women use iron and calcium supplements significantly more frequently than do men, which might be explained by their increased risk of anemia and osteoporosis [26, 27]. Our results support this, given that a significant association between supplement use among women and pregnancy status was found. Evidence from other countries shows that approximately 70%–75% of lactating women use dietary supplements [28–30]. However, in the present study, less than half the women who were breastfeeding were using dietary supplements. Thus, awareness programs for this population are required to increase dietary supplement use during lactation.

In terms of dietary supplement patterns, a single preparation of vitamins, such as beta carotene or vitamins A, B12, B9, B complex, C, D, and E, are the most frequently supplemented in the Saudi population, followed by a single preparation of minerals, such as calcium, zinc, iron, magnesium, potassium, and multivitamins and/or multiminerals. These results are similar to those of Aljaloud et al., who tested the use of dietary supplements among athletes in Saudi Arabia in 2012 and found that vitamin C and calcium had the highest prevalence of use at 82% and 68%, respectively [31].

The main reason for supplement use was to maintain health, similar to the result of a study conducted among university students in Dammam City, which showed that maintaining health was the most common reason for using dietary supplements [6]. Moreover, most of the participants read the attached instructions of these supplements.

Regarding the attitude toward dietary supplements, a recent study conducted in Riyadh found that approximately 89% of participants thought about the risks and side effects of dietary supplements [22]. Another recent report found that more than 70% of female college students used dietary supplements based on medical prescription but reported a lack of proper information and basic knowledge about side effects, which is part of the importance of a physician’s prescription [7]. In this study, the majority of the supplement users were aware of the importance of medical consultation, as well as the negative side effects. They were also aware of the importance of laboratory testing prior to using dietary supplements.

One strength of this study is that, to our knowledge, this is the first to assess the prevalence of dietary supplement use across all regions of Saudi Arabia. The majority of previously conducted studies on this topic were among women only or specific groups such as medical students [7, 20, 21, 23].

### Limitations

This study has limitations. First, it relied on a self-reported questionnaire which may have introduced recall bias. Second, it had a cross-sectional design where self-selection bias is inevitable. Participation may have been more appealing to dietary supplement users than to non-users. Third, the responders in the current study were predominantly from the middle region of Saudi Arabia, and approximately 70% were women, even though the survey link was shared across all regions in Saudi Arabia through various social media platforms and was directed for both sexes. This is due to the use of the snowball sampling technique.

This study measured the frequency of using dietary supplements, not the dosage. A recommendation for future research would be to measure the used dosage of each supplement, brand names, and the duration of use, as well as assessing the diet quality of the participants. In addition, qualitative research to investigate the reasons behind dietary supplement use would provide useful information. It is recommended to use a validated questionnaire to assess dietary supplement use and awareness of use among the study participants. Further investigation is required to estimate the types of herbs used by individuals during illness, the necessity of use, and the reasons behind use. Although this study provides information on the prevalence, patterns, and attitude regarding dietary supplement use, the Saudi population lacks a guide on the appropriate dose and type for each age group, sex, or health status.

In summary, this study is the first to provide data on dietary supplement use by the Saudi population across all regions. Notably, the prevalence of usage was 52%, and maintaining health was the most common reason for using dietary supplements. Furthermore, high education level, working or studying in the medical field, a young age, and pregnancy were significantly associated with dietary supplement use. One of the more important findings of this study was the significant association between lactation and the lack of dietary supplement use. Furthermore, there were no significant differences between those with chronic and non-chronic diseases and there were no significant differences across regions of Saudi Arabia. This study was conducted 6 months prior COVID-19 era. It will be very interesting to re-conducted the study after COVID-19 to look at the difference in the prevalence and the patterns of dietary supplement use in Saudi Arabia.

## Supporting information

S1 Data (XLSX)
